# Electroacupuncture at ST36/SP6 Restores Surgical Stress‐Induced Impairment of Dendritic Cell Maturation via GR/GILZ Signaling in Mice

**DOI:** 10.1002/iid3.70241

**Published:** 2025-07-29

**Authors:** Mengting Jiang, Caixia Liu, Yinzhou Zhang, Sibei Li, Yuhui Li, Chengcheng Zhou

**Affiliations:** ^1^ Department of Anesthesiology, Shanghai General Hospital Shanghai Jiao Tong University of Medicine Shanghai China; ^2^ Guangzhou University of Chinese Medicine Guangzhou Guangdong Province China; ^3^ Department of Anesthesiology, First Affiliated Hospital Guangzhou University of Chinese Medicine Guangzhou Guangdong Province China

**Keywords:** dendritic cells, electroacupuncture, GILZ, GR, perioperative period

## Abstract

**Objective:**

This study aims to examine how surgical stress and electroacupuncture (EA) influence the maturation of splenic dendritic cells (DCs) in mice, with a specific focus on elucidating the mechanisms through which EA influences DC maturation.

**Methods:**

Mice were allocated into four groups: control, model, model + electroacupuncture (EA), and model + non‐electroacupuncture (NEA). For the EA intervention, Zusanli (ST36) and Sanyinjiao (SP6) acupoints received electroacupuncture stimulation, while the NEA group received bilateral stimulation 3 mm adjacent to the midpoint of the line between the tail and the anus. Electroacupuncture was administered 12 h before and during the surgical procedure. Post‐surgery, levels of corticosterone (CORT) and adrenocorticotropic hormone (ACTH) in the serum were quantified using ELISA. The expressions of CD86, MHC‐II, and CD40 on the surface of DCs were assessed via flow cytometry. Quantitative PCR (qPCR) was utilized to detect the mRNA expression levels of glucocorticoid‐induced leucine zipper (GILZ), glucocorticoid receptor (GR), interleukin‐1β (IL‐1β) and interleukin‐6 (IL‐6). Additionally, GR and GILZ protein expression levels were analyzed through Western blot analysis.

**Results:**

Compared to the control group, both the model and NEA groups exhibited reduced CD11c+CD40 + , CD11c+MHC‐II + , and CD11c + CD86 + DC percentages. Conversely, these markers were elevated in the EA group. Additionally, both interleukin‐1β (IL‐1β) and interleukin‐6 (IL‐6) were decreased in the model group and the NEA group, whereas an increment was noticed in the EA group. Notably, both mRNA level and protein expression profile for both GILZ and GR demonstrated a significant increment in the model group and the NEA group, whereas a marked reduction was observed in the EA group. Furthermore, the blockade of GR using RU486 resulted in an increased percentage of CD11c + CD40 + , CD11c + CD86 + , and CD11c+MHC‐II+ DCs post‐surgery.

**Conclusion:**

Application of electroacupuncture (EA) at the ST36 and SP6 acupoints can mitigate the suppressive effects of surgical stress on DC maturation through the GR/GILZ signaling pathway throughout the surgical process.

## Graphical abstract

1. At present, there are very few studies on the mechanism of acupuncture regulating the immune state of DC. This study is the first to explore the mechanism of electroacupuncture affecting the maturation of DC after surgery. 2. This study found that electroacupuncture could effectively reduce the inhibition effect of surgical stress on DC maturation after surgery, reduced the secretion of CORT after surgery, and inhibit the expression of GR, and this therapeutic effect of electroacupuncture was acupoint‐specific. 3. This study found that GR/GILZ signaling pathway is involved in the electroacupuncture regulation of postoperative dendritic cells maturation in mice undergoing surgical stress.

## 1 Introduction

Perioperative immune changes are mainly due to surgical trauma and subsequent neuroendocrine response. A pivotal element in this process is the hypothalamic‐pituitary‐adrenal (HPA) axis. HPA mainly regulates the circulating concentrations of stress hormones, including glucocorticoids (GC) and adrenocorticotropic hormone (ACTH). These hormones are essential for maintaining physiological homeostasis [1]. Surgical procedures and anesthesia provoke a range of metabolic and endocrine responses, leading to widespread immunosuppression immediately following the surgical procedure [2]. Therefore, improving the steady‐state management of HPA axis is very important for surgical‐induced immunosuppression.

Dendritic cells (DCs) are migratory leukocytes specialized in the uptake of antigens, processing and presentation of these antigens to T lymphocytes, its surface expresses CD11c molecule [3]. As crucial mediators in activating naive T lymphocytes, DCs from a vital link between the innate and adaptive immune systems. During periods of homeostasis, DCs remain in an immature state characterized by low MHC‐II expression and minimal or absent expression of costimulatory molecules like CD80, CD86, and CD40 [4]. It can filter extracellular fluid and capture antigens by endocytosis or pinocytosis [5]. Upon maturation, DCs upregulated MHC‐II and costimulatory molecules expression level, including CD80, CD83, CD86, and CD40. This enhanced expression equips them with the capability to activate both Th1 and Th2 cell responses, facilitating effective adaptive immune responses [6].

Surgery can affect the number of DCs in the peripheral circulation. It is found that the number of DCs after surgery is significantly less than that before surgery in both gastrointestinal cancer surgery and endoscopic cholecystectomy [7]. GC released by surgical stress (mammals: cortisol; rodents corticosterone, CORT inhibits the immune response of DCs [8]. GC can activate GR and then induce the expression of GILZ [9]. GILZ has been demonstrated to mediate the effects of GC in human DCs and has increasingly been recognized for its role in regulating DC tolerance for both humans and mice [10].

The acupoints Zusanli (ST36) and Sanyinjiao (SP6) are traditionally employed to modulate immune function. There is substantial evidence indicating that low‐frequency EA stimulation at these specific acupoints can reduce HPA axis hyperactivity [11]. EA offers a nonpharmacological intervention for managing HPA axis disorders resulting from surgical procedures. Previous clinical research has demonstrated that EA stimulation can mitigate the stress response in patients undergoing spinal surgery, underscoring its potential therapeutic benefits in managing perioperative stress [12]. Animal research has revealed that EA stimulation can enhance MHC‐II and CD86 expressions on the DC surface in rats undergoing spinal surgery, thereby mitigating the surgery‐induced inhibition of DC maturation [13].

A surgical model of exploratory laparotomy in mice was established in this study with EA stimulations being administered at the ST36 and SP6 acupoints. We aimed to assess the levels of ACTH and CORT, as well as the expression of CD86, MHC‐II, and CD40 on DCs. Additionally, we measured the relative expression of GR and GILZ to investigate the impact and underlying mechanisms of surgical stress and EA stimulation on the maturation of DCs.

## 2 Materials and Methods

### 2.1 Animals

In this study, male C57BL/6J mice aged between six and eight weeks were utilized for all the experiments. These mice were sourced from the Animal Experiment Center of Guangzhou University of Traditional Chinese Medicine. The mice were housed under barrier conditions with controlled environmental parameters. They had free access to food and water throughout the study, and was not restrained. Experimental procedures began following a 7‐day acclimatization period. All animal experimental protocols involving animals were conducted in strict accordance with the NIH Guidelines for the Care and Use of Laboratory Animals (NIH Publications No. 8023, revised 1978). Approval for this study (approval no. 20210928004) was granted by the Animal Experiment Ethics Committee of Guangzhou University of Chinese Medicine before the commence of the study.

### 2.2 Surgery and Drug Application

The mice were randomly assigned to one of four groups: control, model, EA, and NEA. The mice in the model, EA, and NEA groups underwent the same exploratory laparotomy, as described by Rosczyk et al. [14] before the surgery, these mice were fasted for 12 h and anesthetized using 2% sevoflurane. Expose the midline of the abdomen, cut the skin along the midline, the surgical incision approximately 1 cm long, then cut the fascia, expose the abdominal cavity, and carry out abdominal exploration. During the surgery, it is imperative to maintain the original orientation of the intestines and handle them gently to avoid causing intestinal bleeding or damaging abdominal blood vessels. The duration of the surgery is approximately 10 min. No subsequent interventions (e.g., electroacupuncture treatment or other experimental manipulations) were applied to the model group.

To determine the best time for collecting tissue after surgery, we did the pre‐experiment first, and the mice (*n* = 6) were euthanized at intervals of 2, 6, 12, 24, 48, and 72 h following surgery. To investigate the mechanism of EA's effects, intraperitoneal injections were administered to mice, utilizing either the vehicle (1 mL/kg, consisting of 5% DMSO in 95% PBS) or RU486 (mifepristone, a GR antagonist) dissolved in the vehicle at a dosage of 20 µg/kg. These injections were administered 12 h before and immediately after the surgery.

### 2.3 EA Treatment

**Figure 1:**
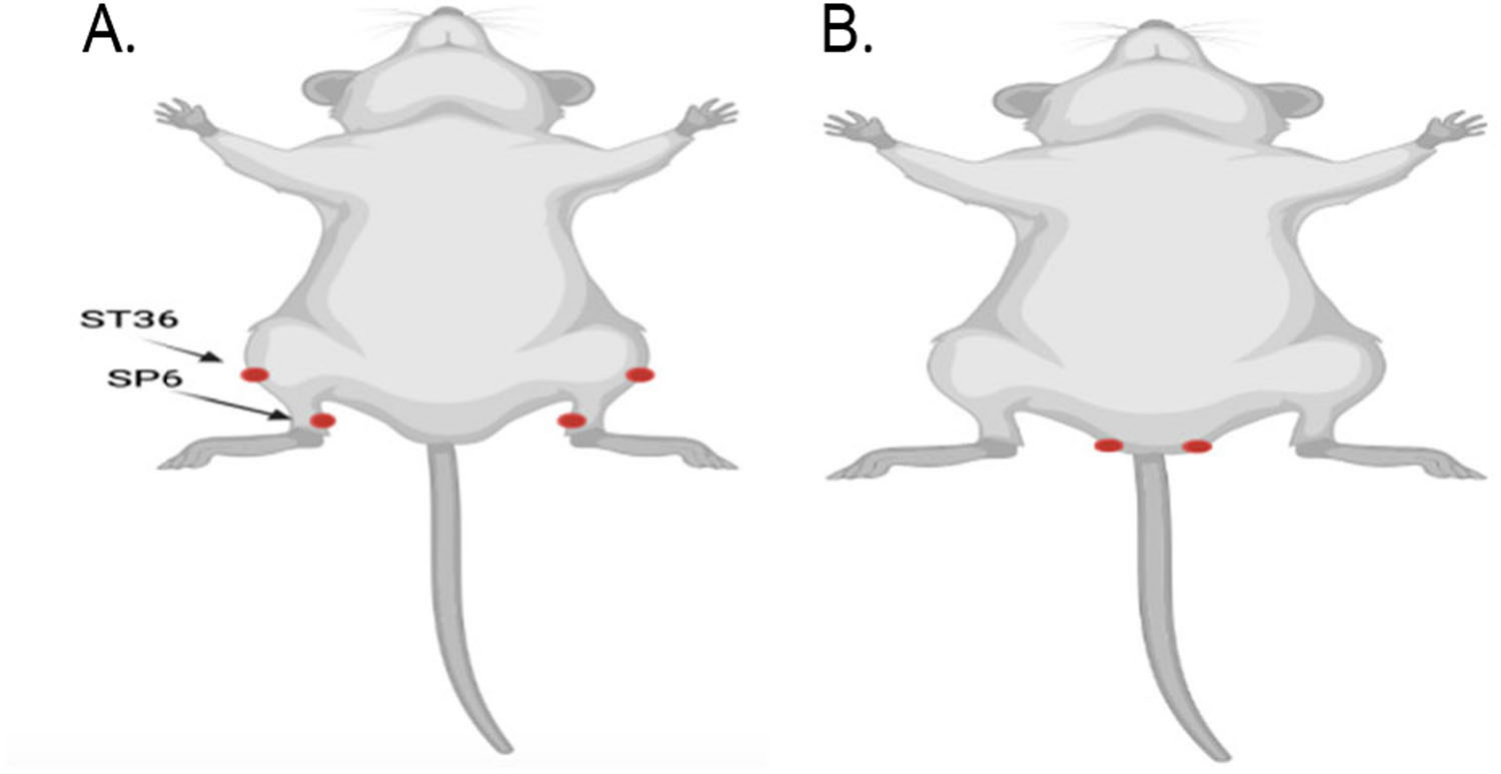
Presents a detailed topographical diagram illustrating the specific points on mice that were stimulated during acupuncture treatments in the study. (A) the diagram depicts the Electroacupuncture (EA) group. In this setup, the mice received bilateral stimulation at two acupuncture points: “ST36” (located near the knee joint) and “SP6” (situated on the lower leg, above the ankle). These points are traditionally targeted in acupuncture to promote healing and reduce inflammation. (B) shows the Non‐Electroacupuncture (NEA) group, where the mice were stimulated at nonspecific points, termed non‐acupoints. These points do not correspond to any traditional acupuncture points and are used to serve as controls to evaluate the effectiveness of acupuncture at designated therapeutic points. This comparison helps in assessing the specific impact of targeted acupuncture point stimulation vs. random point stimulation.

The application of EA was conducted in accordance with the methodology described by Ding et al. [15] Mice in the EA and NEA groups received EA stimulation. The stimulation sites of EA group were bilateral “ST36” (located at the lower lateral side of knee joint and 3.5 mm below fibular capitulum) and “SP6” (located at about 5 mm above the tip of medial malleolus of hind limb). For the NEA group, the stimulation sites were located bilaterally, 3 mm next to the mid‐point on the line between the mouse tail and the anus, targeting the hip muscles. The stimulation intensity was set at 1 mA, utilizing an alternating dense‐sparse frequency sequence: 2 Hz for 1.05 s and 15 Hz for 2.85 s, alternating. Electroacupuncture (EA) treatment was administered 12 h before surgery for a duration of 30 min and again 10 min before the surgery, continuing until the conclusion of the surgical procedure Figure 1.

### 2.4 Tissue Collection

After surgery, mice in each group (*N* = 6) were euthanized through cervical dislocation, followed by exsanguination with normal saline. Their spleens were promptly harvested. A portion of the tissue was analyzed using flow cytometry. The remaining tissue was then promptly frozen in liquid nitrogen and stored at −80°C until further processing.

### 2.5 ELISA

At the time of euthanasia, blood samples were obtained through orbital enucleation. These samples were then subjected to centrifugation to separate the serum. Quantification of corticosterone (CORT) and adrenocorticotropic hormone (ACTH) levels was performed using ELISA kits acquired from Elabscience (Wuhan, China) and Cayman Chemical (Ann Arbor, Michigan, USA), respectively, adhering strictly to the manufacturers' protocols. The optical density (OD) values of these individual wells was then measured using a UV microplate reader. All serum samples were assayed in a single batch to ensure consistency, and each sample was analyzed in duplicate.

### 2.6 Flow Cytometry

The spleens were harvested and placed in a culture dish containing pre‐cooled PBS (Gibco, Gaithersburg, Maryland, USA). The tissues were dissociated using a 200‐mesh cell sieve (SORFA, Zhejiang, China) while being simultaneously rinsed with PBS to collect the splenocyte suspension. The resulting splenocyte suspension was then passed through a 200‐mesh cell sieve again and centrifuged at 1700 rpm for 5 min. The supernatant is then discarded before the addition of 5 mL of red blood cell lysis buffer (Solarbio, Beijing, China), followed by direct ice incubation for 10 min. Then, the sample is subjected to another centrifugation, where the supernatant was then discarded, and the cells were resuspended in 2 mL of PBS.

For flow cytometry, an aliquot of 100 µL of the splenocyte suspension was transferred into a flow cytometry tube, to which the following fluorochrome‐conjugated antibodies were added: PE‐MHC‐II, PE‐Cy7‐CD40, APC‐CD86, and FITC‐CD11c, (Biolegend, California, USA). The mixture was thoroughly mixed and incubated on ice for 30 min under dark conditions. Subsequently, the cells were washed twice with 2 mL of PBS, with the supernatant being discarded after each wash. The cells were finally resuspended in 500 µL of PBS. Approximately 10^6^ cells were analyzed by flow cytometry. Our gating strategy consists of successive double exclusion steps (FSC‐A vs. FSC‐H), by selecting CD11c+ dendritic cells, and surface labeling analysis using homotype and FMO control to determine positive thresholds (CD40, CD86, MHC‐II).

### 2.7 Quantitative Polymerase Chain Reaction (qPCR)

For the analysis of mRNA, Trizol reagent was utilized to extract total RNA from the spleen (Invitrogen, California, USA), in accordance with the protocols provided by the manufacturer. The assessment of RNA integrity and purity was performed using a spectrophotometer. Reverse transcription was then carried out, with the resultant complementary DNA (cDNA) being kept at −20°C for subsequent experiments. Quantitative PCR (qPCR) was conducted by amplifying 1 µg of cDNA with the SYBR Green Pro Taq HS qPCR kit (Accurate Biology). The thermal cycling protocol consisted of an initial denaturation phase that took place at a temperature of 95°C for a duration of 30 s, and 40 subsequent cycles at a temperature of 95°C for a duration of 5 s, followed by 60°C for another 30 s. Purity and specificity of the amplified products were verified through a melting curve analysis post‐amplification. Primers were custom synthesized by Shanghai Bioengineering Company for IL‐6 (Forward: CTCCCAACAGACATGTCTATAC, Reverse: CCATTGCACAACTATTTTCTCA), IL‐1β (Forward: GCTTCCAAACCTTTGACCTG, Reverse: CTGTTGTTTCCCAGGAAGAC), GR (Forward: ACTCCAAAGAATCCTTAGCTCC, Reverse: TATACAAGTCCATCACGCTTCC), GILZ (Forward: CCCTAGACAACAAGATTGAGC, Reverse: GAATCTGCTCCTTTAGGACCTC), and β‐actin (Forward: GTCGTACCACAGGCATTGTGATGG, Reverse: GCAATGCCTGGGTACATGGTGG). All assays were conducted in sets of three, and the expression levels of mRNA were calculated using the $2^{-∆∆C_{T}}$ method, ensuring normalization against β‐actin to serve as the reference standard.

### 2.8 Western blot

After weighing part of the spleen tissue, add RIPA buffer (Beyotime, Shanghai, China) within Phenylmethanesulfonylfluoride (Beyotime) according to the spleen mass and put it into a homogenizer for full lysis. The BCA protein assay kit obtained from Beyotime was utilized in accordance to the manufacturer's calibration instructions to determine protein concentration. Denaturation of the proteins was achieved through heating at 100°C for 10 min in a 5× Loading Buffer solution (Beyotime). The denatured proteins were then separated using 10% SDS‐PAGE gel and subsequently transferred to polyvinylidene fluoride (PVDF) membranes (Millipore, Massachusetts, USA). To block nonspecific binding sites, the membranes were incubated with 5% skimmed milk for 1 h at room temperature, followed by overnight incubation with primary antibodies at 4°C. (GR: 1:500, Affinity, Changzhou, China; GILZ: 1:500, Protientech, Wuhan, China; α‐Tubulin: 1:2000, Protientech).

After primary antibody incubation, the membranes were then washed three times with TBST buffer, followed by 1‐h incubation with horseradish peroxidase (HRP)‐conjugated secondary antibodies at a 1:5000 dilution (Proteintech) at ambient temperature. Protein signals were visualized with the aid of an ECL hypersensitive chemiluminescence kit (Millipore). The emitted chemiluminescent signals were captured and quantified using Image Lab software (Bio‐Rad, USA). Signal intensity was measured in arbitrary densitometric units and normalized to α‐Tubulin, which served as an internal standard, to ensure accuracy in protein quantification.

### 2.9 Statistical Analysis

Statistical analyses were conducted on the SPSS 26 statistics software (IBM Corporation, NY, USA). Results are expressed as the mean values with standard deviation (SD). Group differences were analyzed using one‐way analysis of variance (ANOVA). For post‐hoc multiple comparisons, either the Least Significant Difference (LSD) *t*‐test or the Games‐Howell test was employed. A *p*‐value less than 0.05 was considered statistically significant.

## 3 Results

### 3.1 The Expression of CD86 and MHC‐II on DCs in the Control Group and Various Time Points Post‐Surgery

Flow cytometry was employed to examine the effects of surgical stress on CD86 and MHC‐II expression on DCs in mice. The percentage of CD11c + CD86+ cells exhibited a notable reduction at 12 and 24 h after surgery compared to the control group (*p* < 0.05). While there was a trend toward a decrease in the percentage of CD11c+CD86+ cells at 2, 6, 48, and 72 h after surgery, these changes did not reach statistical significance (*p* > 0.05). Notably, the percentage of CD11c+CD86+ cells at 12 h post‐surgery was also significantly lower compared to the percentages at 2‐, 6‐, and 48‐h post‐surgery (*p* < 0.05).

**Figure 2:**
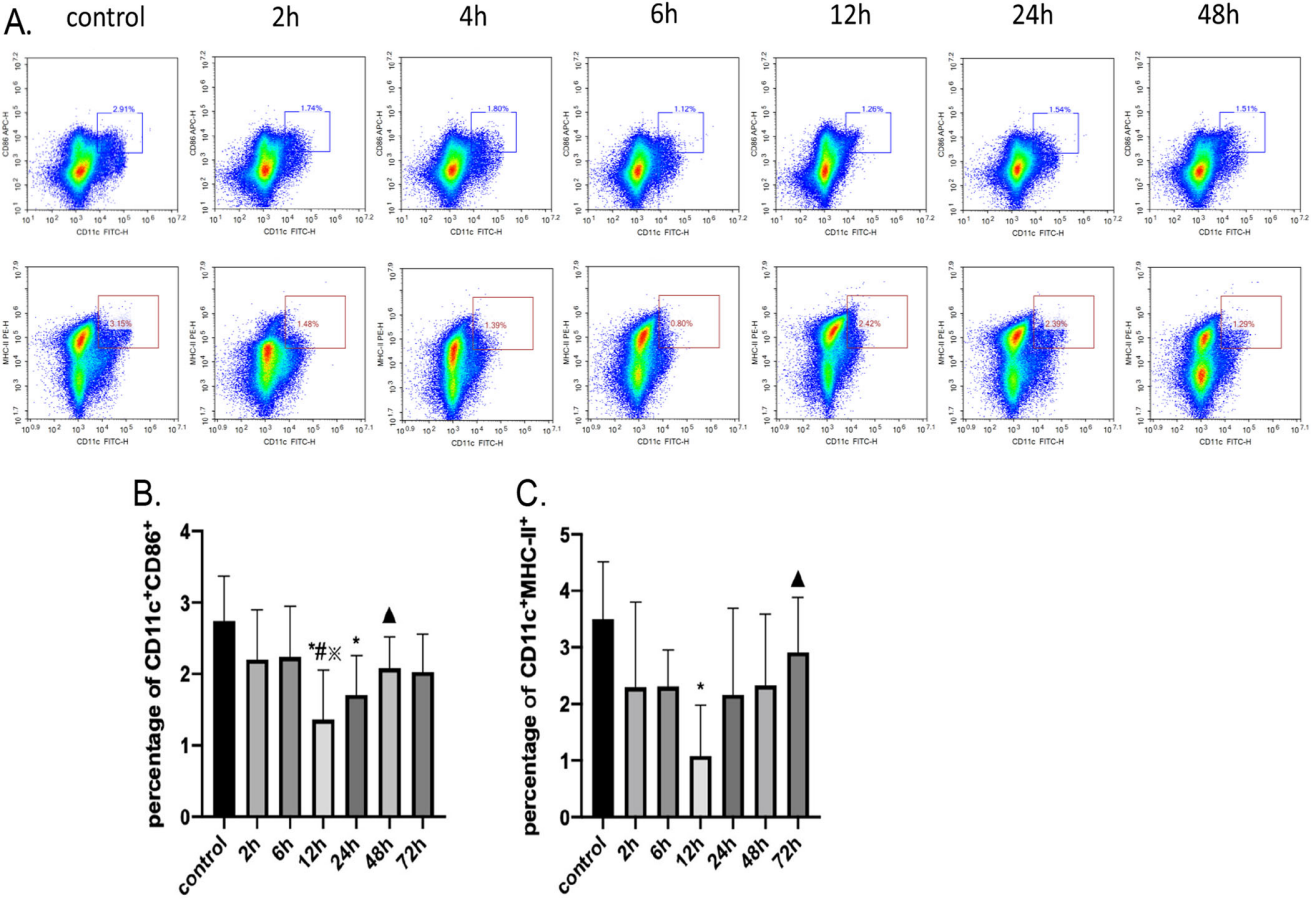
Illustrates the variations in the expression levels of CD86 and MHC‐II on dendritic cells (DCs) at different time points following surgery, based on flow cytometry testing. The expression levels are compared across several intervals—control (presurgery), and 2, 6, 12, 24, 48, and 72 h post‐surgery. (A) presents the flow cytometry results, depicting the quantitative changes in the expression levels over time. This data helps in understanding the immune response dynamics post‐surgery. (B) focuses on the proportion of cells in the spleen expressing both CD11 and CD86 markers. (C) Quantifies the cells co‐expressing CD11 and MHC‐II. These markers are crucial for the activation and antigen‐presenting functions of dendritic cells, which play a key role in initiating immune responses. The data in the figure are presented as the mean ± standard deviation (SD) for six samples (*N* = 6). Statistical significance is indicated by symbols: an asterisk (*) denotes a significant difference from the control group, a hash (#) marks significance in comparison with the 2‐h post‐surgery group, a star (※) indicates a significant difference from the 6‐h post‐surgery group, and a triangle (▲) highlights differences compared to the 12‐h post‐surgery group, all at a *p*‐value of less than 0.05.

Similarly, at 12 h post‐surgery, a significant decrease in CD11c+MHC‐II+ cell percentage was noted when compared to the control group (*p* < 0.05). Although there was a downward trend in the percentage of CD11c+MHC‐II+ cells at 2‐, 6‐, 24‐, 48‐, and 72‐h post‐surgery, the reductions at these time points did not reach statistical significance (*p* > 0.05). Additionally, the percentage of CD11c+MHC‐II+ cells at 12 h postoperation was notably lower than that observed at 72 h after surgery (*p* < 0.05). Collectively, these data demonstrate that surgical stress transiently downregulates DC maturation markers, with the most pronounced suppression of both CD86 and MHC‐II expression occurring at 12 h post‐surgery, suggesting a time‐dependent impairment of DC antigen‐presenting capacity during the acute postoperative phase. These results are presented in Figure 2.

### 3.2 Impact of EA on CD40, CD86, and MHC‐II Expression on DCs in Surgically Stressed Mice

**Figure 3:**
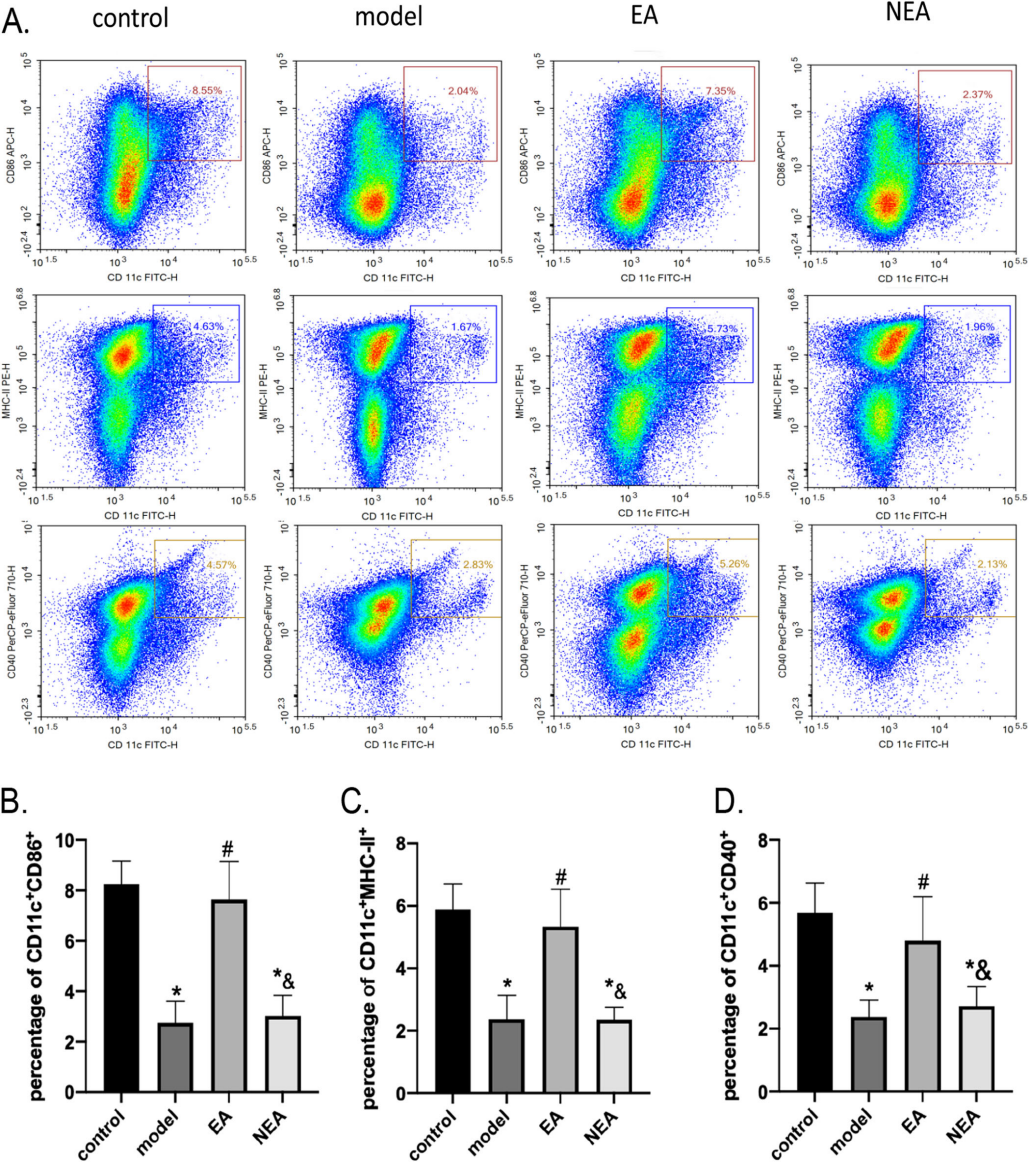
Displays the expression levels of CD86, MHC‐II, and CD40 on dendritic cells (DCs) across different experimental groups: control, model, Electroacupuncture (EA), and Non‐Electroacupuncture (NEA). This is analyzed using flow cytometry to compare how different treatments affect immune cell behavior. (A) presents the flow cytometry test results, illustrating the overall expression levels of the markers CD86, MHC‐II, and CD40 on DCs. (B), (C), and (D) provide detailed percentages of dendritic cells in the spleen expressing combinations of surface markers: CD11 and CD86 (b), CD11 and MHC‐II (c), and CD11c and CD40 (d), respectively. The data across these panels are presented as mean ± standard deviation (SD) for six replicates (*N* = 6). Statistical significance is denoted with different symbols to indicate comparisons: an asterisk (*) shows a significant difference from the control group, a hash (#) indicates significant differences from the model group, and an ampersand (&) denotes significant differences when compared with the EA group, all with a *p*‐value of less than 0.05.

Flow cytometry was used to assess the impact of EA on DCs, especially on the expression levels of CD40, CD86, and MHC‐II, in mice subjected to surgical stress. Postoperative evaluations revealed differences of statistical significance in the expression of these markers among the various groups. The model and the NEA groups exhibited lower percentages of CD11c + CD40 + , CD11c + CD86 + , and CD11c+MHC‐II+DCs compared to the control (*p* < 0.05). Conversely, the EA group did not show significant differences from the control group in these percentages (*p* > 0.05). Furthermore, the EA group demonstrated notably higher percentages of CD11c + CD40 + , CD11c + CD86 + , and CD11c+MHC‐II+ DCs relative to the model group (*p* < 0.05). Additionally, the NEA group had significantly lower percentages of these markers (*p* < 0.05). These findings indicate that EA, but not NEA, effectively preserves the expression of DC maturation markers in surgically stressed mice, restoring their levels to near‐normal values and highlighting the specificity of EA in mitigating surgery‐induced DC functional impairment. These findings are detailed in Figure 3.

### 3.3 Impact of EA on Serum CORT and ACTH Levels in Surgically Stressed Mice

**Figure 4:**
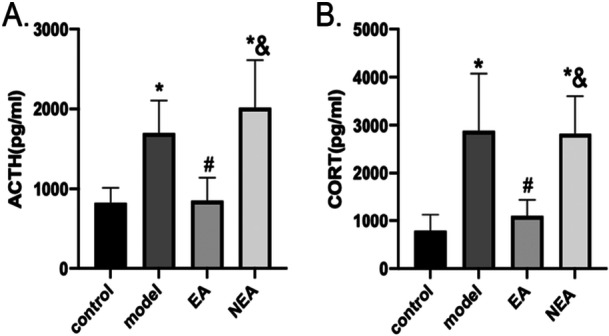
Illustrates the levels of Adrenocorticotropic Hormone (ACTH) (A) and Cortisol (CORT) (B) in the blood of mice across four different groups: control, model, Electroacupuncture (EA), and Non‐Electroacupuncture (NEA). The data in both panels are presented as the mean ± standard deviation (SD) for a sample size of six (*N* = 6). The statistical significance is marked by symbols where an asterisk (*) indicates a significant difference compared to the control group, a hash (#) indicates a significant difference from the model group, and an ampersand (&) signifies differences relative to the EA group. All statistical comparisons are made at a significance level of *p* < 0.05.

To evaluate the influence of EA on serum levels of CORT and ACTH in surgically stressed mice, ELISA analyses were performed. The results demonstrated statistically significant variations between the experimental groups. Both the model and the NEA groups exhibited markedly elevated levels of CORT and ACTH compared with control (*p* < 0.05). In contrast, the EA group did not exhibit differences of statistical significance (*p* > 0.05). Additionally, the EA group demonstrated significantly reduced CORT and ACTH levels than the model group (*p* < 0.05), whereas the NEA group recorded significantly higher levels of CORT and ACTH compared to the EA group (*p* < 0.05). These data collectively suggest that electroacupuncture (EA) selectively suppresses surgery‐induced hyperactivation of the HPA axis, as evidenced by normalization of serum CORT and ACTH levels, whereas non‐acupoint stimulation (NEA) fails to counteract the surgical stress‐triggered endocrine dysregulation. These findings are illustrated in Figure 4.

### 3.4 Effects of EA on IL‐1β and IL‐6 Cytokine Expression in Surgically Stressed Mice

**Figure 5:**
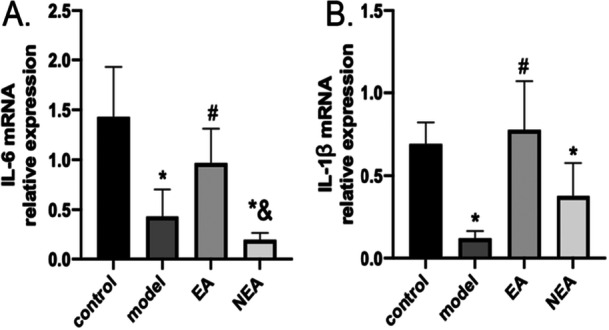
Presents the mRNA expression levels of two key inflammatory cytokines, Interleukin‐6 (IL‐6) (A) and Interleukin‐1 beta (IL‐1β) (B), in the spleen across four different experimental groups: control, model, Electroacupuncture (EA), and Non‐Electroacupuncture (NEA). The data for both panels are reported as the mean ± standard deviation (SD) for six samples (*N* = 6). The statistical significance of differences in expression levels is indicated by the following symbols: an asterisk (*) signifies a significant difference compared to the control group, a hash (#) signifies a significant difference from the model group, and an ampersand (&) denotes a significant difference when compared with the EA group, all at a *p*‐value of less than 0.05.

qPCR was utilized to assess how EA affects the mRNA expression levels of cytokines, specifically IL‐1β and IL‐6, in surgically stressed mice. The results demonstrated statistically significant differences across the groups. The model and the NEA groups exhibited significantly decreased levels of IL‐1β and IL‐6 mRNA relative to the control group (*p* < 0.05). Conversely, the EA group showed no differences of statistical significance from the control group in these cytokine levels (*p* > 0.05). Furthermore, the EA group demonstrated significantly higher IL‐1β and IL‐6 mRNA expression levels than the model group (*p* < 0.05). Additionally, the expression levels of IL‐6 mRNA were greatly decreased in the NEA group compared to the EA group (*p* < 0.05). Together, these data suggest that electroacupuncture (EA) prevents surgery‐induced suppression of IL‐1β and IL‐6 mRNA expression, maintaining cytokine levels comparable to baseline controls, while non‐acupoint stimulation (NEA) fails to restore these cytokines and further exacerbates IL‐6 reduction. Please see Figure 5 for details.

### 3.5 Effect of EA on GR and GILZ Expression in Surgically Stressed Mice

**Figure 6:**
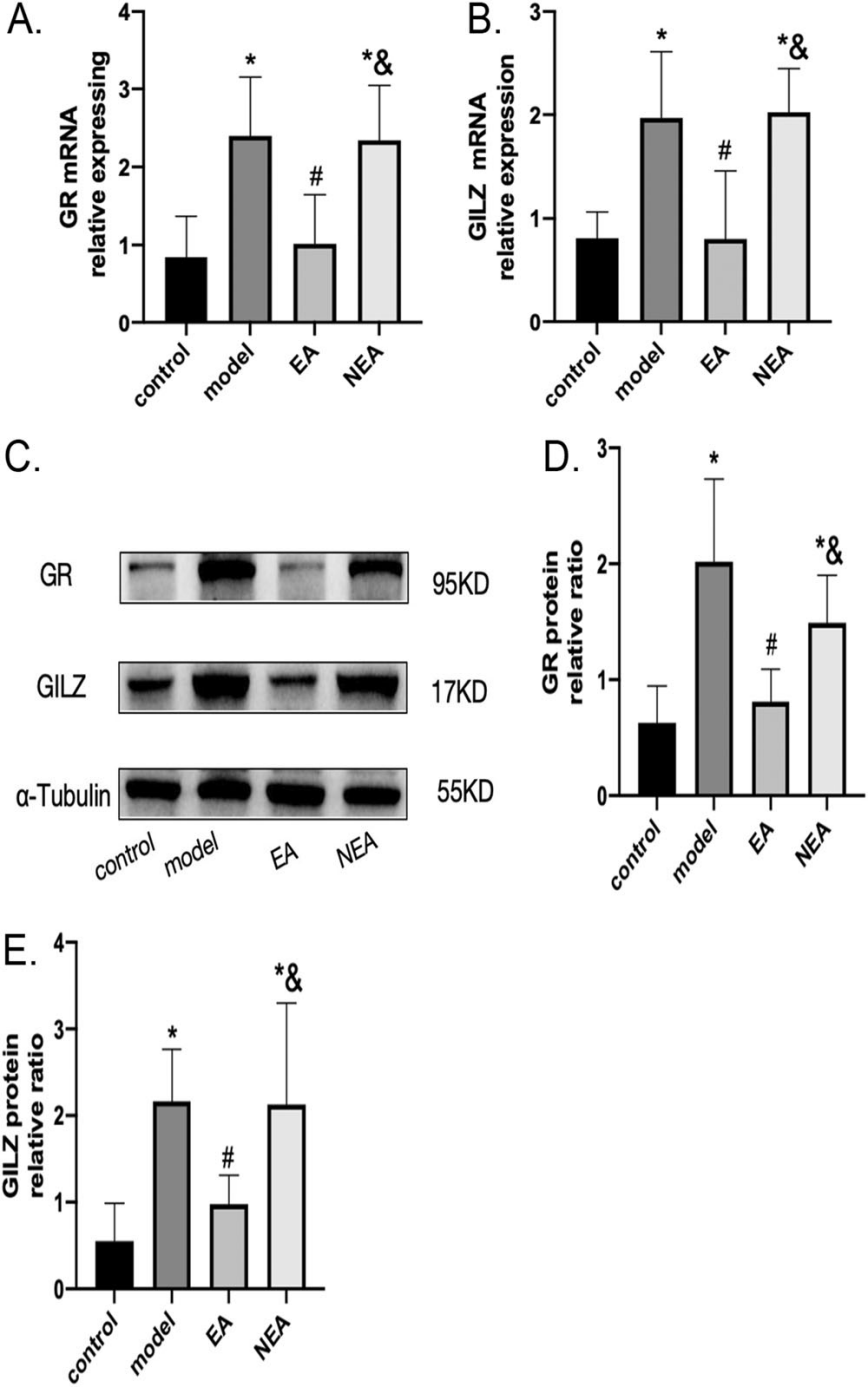
Presents the expression levels of glucocorticoid receptor (GR) and glucocorticoid‐induced leucine zipper (GILZ) in the spleen across different experimental groups: control, model, Electroacupuncture (EA), and Non‐Electroacupuncture (NEA). Both GR and GILZ play significant roles in the regulation of immune responses and inflammation through their interaction with glucocorticoids. (A) details the mRNA expression levels of GR. (B) shows the mRNA expression levels of GILZ, (C) presents representative bands from a Western blot analysis, visually illustrating the protein expression of GR and GILZ in the spleen. Panels (D) and (E) provide the quantification of protein expression for GR and GILZ, respectively. These panels plot the densitometry results from the Western blot. The data across all panels are presented as the mean ± standard deviation (SD) for a sample size of six (*N* = 6). Statistical significances are denoted by an asterisk (*) indicating a significant difference from the control group, a hash (#) showing a significant difference from the model group. An ampersand (&) indicating a significant difference compared to the EA group.

To investigate the involvement of GR and GILZ in the immunosuppression of DCs and the potential role of EA in reversing this immunosuppression, the expression levels of GR and GILZ in the spleen were assessed. Statistically significant differences in GR and GILZ mRNA and protein expression were noted across the different groups following surgery. Both the model and the NEA groups showed significantly increased levels of GR and GILZ mRNA and protein expression levels compared to control (*p* < 0.05), whereas the EA group did not demonstrate any notable differences from control (*p* > 0.05). Notably, the EA group exhibited a notable decrease in the expression levels of mRNA and proteins for both GR and GILZ in comparison with the model group (*p* < 0.05). Furthermore, the expression levels of GR and GILZ in the NEA group were significantly higher than those in the EA group (*p* < 0.05). These results suggest that electroacupuncture (EA) selectively downregulates the glucocorticoid receptor (GR) and its downstream effector GILZ in surgically stressed mice, thereby counteracting the surgery‐induced overexpression of this immunosuppressive pathway. These results are presented in Figure 6.

### 3.6 Expression of Dendritic Cell Markers and GR, GILZ in the Spleen After Drug Intervention

**Figure 7:**
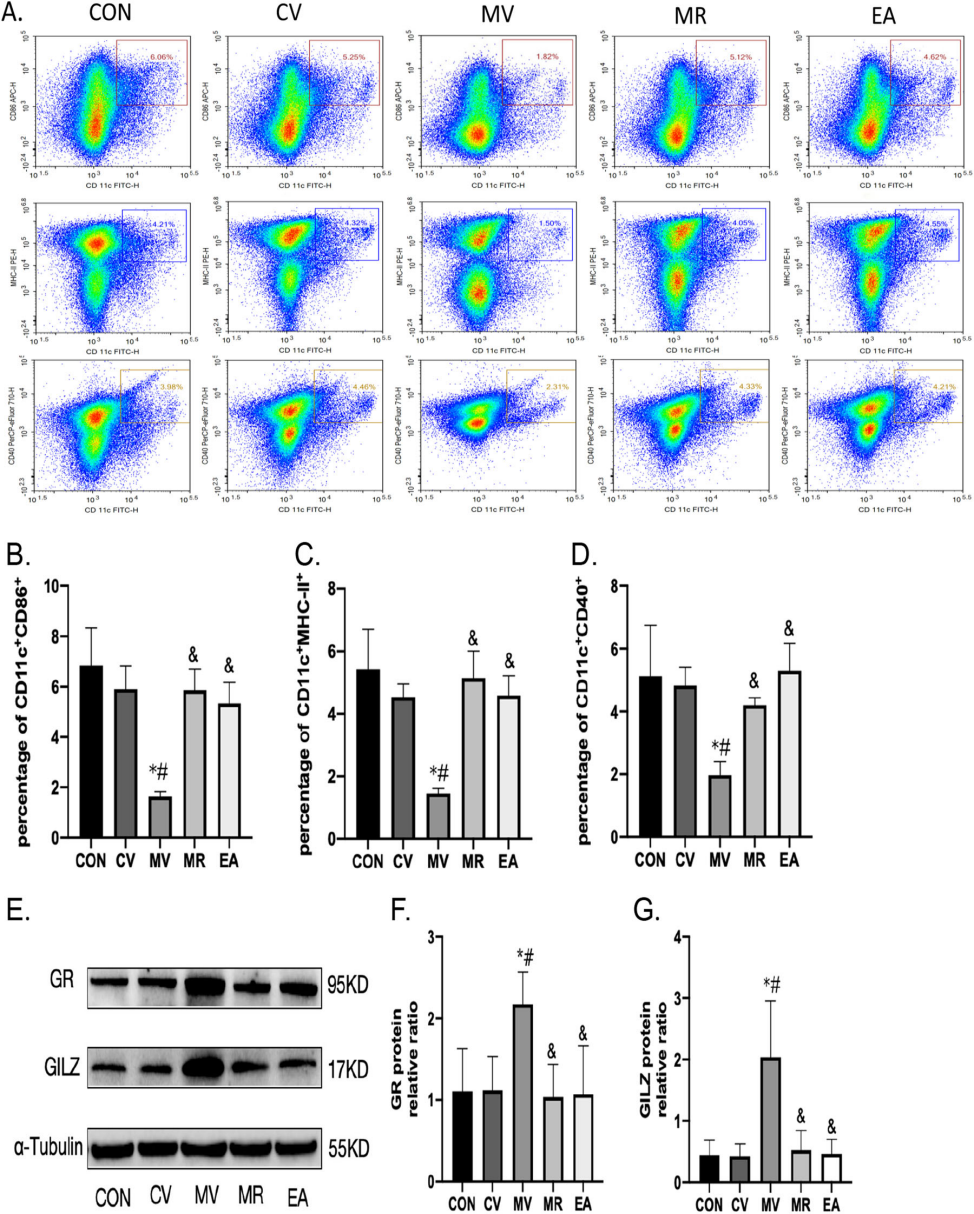
Presents an extensive analysis of the expression levels of several key immunological markers and proteins in the spleen across various experimental groups: control (CON), vehicle‐treated control group (CV group), vehicle‐treated model group (MV group), model+RU486 group (MR group), and Electroacupuncture (EA). (A) displays the results of flow cytometry testing, showcasing the overall expression levels of CD86, MHC‐II, and CD40 on dendritic cells (DCs). (B) illustrates the proportion of cells in the spleen expressing CD86 in combination with CD11c, (C) details the proportion of cells that are double‐positive for CD11c and MHC‐II, indicating their role in antigen presentation to T cells. (D) the proportion of spleen cells expressing both CD11c and CD40. (E) representative Western blot bands for GR and GILZ proteins. (F) and (G) provide quantitative analysis of the protein expression levels for GR and GILZ, respectively, as derived from densitometry of the Western blot bands. The data across these panels are presented as the mean ± standard deviation (SD) for six samples (*N* = 6). Statistical significance is indicated as follows: An asterisk (*) signifies a significant difference from the control group (CON). A hash (#) denotes a significant difference from the CV group. An ampersand (&) marks a significant difference when compared to the MV group.

This segment of the study examined how blocking the glucocorticoid receptor (GR) with the antagonist RU486 affects DC maturation, specifically looking at the surface markers CD40, CD86, and MHC‐II, and the protein expression of GR and GILZ in the spleen following drug administration. Comparing the control group (CON group) and the vehicle‐treated control group (CV group), there were no differences of statistical significance in the percentages of CD11c + CD40 + , CD11c + CD86 + , and CD11c+MHC‐II+ cells or the expression levels of GR and GILZ proteins (*p* > 0.05). However, inhibiting GR with RU486 resulted in a marked increase in these DC surface markers and a decrease in GR and GILZ protein levels group compared to the vehicle‐treated model group (MV group) (*p* < 0.05). Furthermore, when comparing the EA group with the model+RU486 group (MR group), there were no differences of statistical significance in the expression of these markers (CD11c + CD40 + , CD11c + CD86 + , andCD11c+MHC‐II + ) or proteins (GR and GILZ) (*p* > 0.05). These data collectively suggest that pharmacological blockade of the glucocorticoid receptor (GR) reverses surgery‐induced DC maturation suppression, potentially through the GR/GILZ axis, and that electroacupuncture (EA) achieves comparable restoration of DC function to RU486 intervention, implicating shared downstream regulatory mechanisms between EA and glucocorticoid signaling pathways. These findings are detailed in Figure 7.

## 4 Discussion

The exploratory laparotomy stress model is widely used. Its main advantages are less surgical trauma, less impact on the vital signs of mice, as well as minimal damage to other organs, ensuring stability in experimental conditions [14]. In this study, we found that the expression levels of MHC‐Ⅱ and CD86 on DCs began to decline at 2 h after surgery and didn't return to the preoperative levels 72 h after surgery, indicating that the surgery under general anesthesia will significantly inhibit the maturation of DCs. Notably, the suppression of MHC‐Ⅱ and CD86 was more pronounced at 12 h after surgery. Therefore, the subsequent study was chosen to be conducted at 12 h after surgery.

Perioperative immune changes are primarily driven by surgical trauma and the resulting neuroendocrine responses [2]. A key component of this stress response is the activation of the HPA axis, characterized by increased production of pituitary ACTH, which is then followed by the adrenal cortex releasing GC. This process is essential to the modulation of immune functions during and after surgical procedures [16]. ACTH and GC can last for several days postoperatively, and are directly proportional to the severity of surgical stress [17]. In this study, the observed significant elevations in serum CORT and ACTH levels following surgery indicate that the HPA axis was overactivated as a result of the surgical procedure. DCs are critical to the immune landscape, functioning as highly effective antigen‐presenting cells that initiate adaptive immune responses when mature. Mature DCs are characterized by high expression levels of MHC‐II molecules, with the accompaniment of classic costimulatory molecules such as CD40, CD80, and CD86. Depending on the surrounding environmental factors, DCs can either induce an immune response or promote immune tolerance [8]. GC has immunosuppressive effects on DCs. Its effect on DCs is to inhibit the transformation of immature DCs to mature DCs, inhibit antigen processing and presentation, interfere with the migration of mature DCs, and block the production of cytokines in mature DCs, so that GC will induce tolerance DCs [8]. CORT produced by stress will interfere with the activation of T cells by DCs, and even impair the antitumor immunity [9].

In this study, the significantly reduced surface levels of CD40, CD86, and MHC‐II on postoperative DCs indicate inhibition of DC as a consequence of the surgical intervention. GR, acting as a transcription factor that suppresses inflammatory cytokine and chemokine expressions by interacting with and suppressing the activity of specific transcription factors [18]. The anti‐inflammatory characteristics, alongside the immunosuppressive properties of GCs, primarily operate through the modulation of GR and GILZ. GILZ has been shown to be a crucial factor in enabling GC to induce the production of tolerogenic DCs [10]. While this study demonstrates that acute surgical stress‐induced GC elevation suppresses DC maturation via the GR/GILZ pathway, it is crucial to recognize the time‐ and dose‐dependent duality of GC actions. Prolonged GC exposure (e.g., chronic postoperative stress or exogenous GC therapy) may elicit pro‐inflammatory effects through GR desensitization or noncanonical pathways (e.g., MAPK/JNK), such as chemokine (CXCL1, IL‐8) secretion or neutrophil activation [19, 20]. In our model, EA intervention reduces GC levels (serum CORT) and GR/GILZ signaling, potentially mitigating both acute immunosuppressive and latent chronic pro‐inflammatory risks of GCs. However, the observation window (24–72 h post‐surgery) may be insufficient to capture long‐term GC effects. Future studies with extended timelines are needed to clarify EA's regulatory capacity over GC bidirectional actions.

In mice, GR inhibits antigen‐stimulated inflammation mediated by macrophages, DCs, and epithelial cells. This suppression extends to impairing cellular immune responses via the downregulation of IFN‐γ and the inhibition of Th1, Natural Killer (NK), and CD8 + T cells. Although these immunosuppressive effects protect against the lethality of excessive inflammation, they also increase susceptibility to infections and cancer [21]. In this study, a significant elevation in both protein and mRNA levels of GR and GILZ was observed in the spleens of mice subjected to surgery. Concurrently, the expression of pro‐inflammatory cytokines IL‐1β and IL‐6 was markedly reduced. These findings suggest that surgical stress may produce immunosuppressive effect on DCs through GR/GILZ pathway.

The immunomodulatory effects of EA are widely acknowledged. EA helps maintain homeostasis by enhancing the body's capacity to manage stress responses. Stimulation at the ST36 and SP6 acupoints has been shown to regulate hypothalamic peptides, contributing to this stress adaptation and immune regulation [22]. EA at ST36 has also been shown to inhibit the over‐activation of the HPA axis [23]. Additionally, EA has been shown to mitigate postoperative hyperglycemia by suppressing the release of ACTH and enhancing immune functionality during general anesthesia [24]. EA at ST36 and SP6 points can enhance the cytotoxicity of NK cells in postoperative patients besides improving the immune response in individuals diagnosed with liver and gastric cancers [25, 26]. Our previous clinical studies revealed that 2/15 Hz EA stimulation of SP6 and ST36 during the perioperative period more effectively reduces surgical stress than 2/100 Hz EA stimulation. Moreover, it increased the expression of MHC‐II and CD86 in rats undergoing posterior spinal surgery [12, 13]. These findings informed the selection of SP6 and ST36 for the current study. EA has also been documented to trigger neuropeptide release through peripheral electrical stimulation at varying frequencies [27]. Applying different EA stimulation parameters to acupoints will have different physiological effects on the body [28]. It is generally believed that the use of 2 Hz EA stimulation can increase endorphin and enkephalin release from the central nervous system to play an analgesic role, while 100 Hz EA stimulation of acupoints will more increase the release of Dynorphins to play an analgesic role [27]. When 15 Hz EA stimulation is used to acupoints, the analgesic effect of the two mechanisms is equal [29]. Our previous research found that during surgery, 2/15 Hz EA stimulation of SP6 and ST36 can more effectively mitigate surgical stress than 2/100 Hz EA stimulation, which is reflected in the increase of serum cortisol in surgical patients receiving 2/15 Hz EA stimulation is significantly lower than that in patients receiving 2/100 Hz EA stimulation of acupoints [30, 31], and 2/15 Hz EA stimulation can effectively promote CD86 and MHC‐Ⅱ expressions on DC surface after spinal surgery [13]. Therefore, 2/15 Hz EA stimulation was used in this study.

In this study, EA at the SP6 and ST36 points significantly reduced levels of CORT and ACTH in mice serum after surgery, suggesting that EA inhibits HPA axis overactivation caused by surgical stress, thereby alleviating the associated stress response. Additionally, the EA group showed substantial elevation of CD40, CD86, and MHC‐II expression, along with elevated IL‐1β and IL‐6 expression levels, indicating that EA stimulation effectively mitigates the suppressive effects of surgical stress on DC maturation. Moreover, the expression of GR and GILZ mRNA and proteins were notably reduced in the spleens of the EA group compared to the model group, suggesting that EA may regulate DC maturation under surgical stress via the GR/GILZ signaling pathway. In the experiment of studying the mechanism of EA, it is also essential to explain the specificity of acupoints by comparing acupoints with non‐acupoints. In this study, bilateral 3 mm hip muscles next to the mid‐point of the line between the mouse tail and the anus were chosen as the distal non‐acupoint sites, following the methodology described by Ling et al. [32] Serum concentrations of CORT and ACTH were significantly elevated in the NEA group, indicating that NEA stimulation did not effectively reduce the stress response induced by surgery. Additionally, CD40, CD86, and MHC‐II expression on DC surfaces was significantly decreased, along with a notable reduction in IL‐6 and IL‐1β levels. This suggests that NEA stimulation failed to alleviate the immunosuppressive effects of surgical stress on DCs. These findings highlight the acupoint specificity of EA in regulating stress and immune responses. RU486 is a competitive GR inhibitor and the first and only available GR antagonist [33]. It was found that RU486 did not alter plasma CORT levels in stressed rats, but it was able to attenuate the stress‐induced activation of the glucocorticoid receptor (GC‐GR) signaling pathway in the heart and adipose tissue [34]. In this study, comparisons between the control and the vehicle‐treated control group demonstrated no differences of statistical significance in CD40, CD86, MHC‐II, CD40, GR, and GILZ expression, indicating that the vehicle had no impact on these measured indices. In the mode l+ vehicle group, protein expression of GR and GILZ was significantly elevated, along with a notable decrease in the CD40, CD86, and MHC‐II expression. Conversely, the model+RU486 group showed the opposite pattern, indicating the involvement of the GR/GILZ pathway in the suppressive effects of surgical stress in relevance to DC maturation. While current research on postoperative immunosuppression primarily focuses on NK cells, B cells, and T cells, our study emphasizes DCs, the key antigen‐presenting cells that initiate the adaptive immune response, which are rarely discussed in this context. However, DCs needs to be further selected from the spleen to explore the specific effect of EA on DCs from GR/GILZ signal transduction pathway at the cellular level.

Of course, our research still has some shortcomings, such as this study lies in its exclusive focus on male mice, which precludes an assessment of potential sex‐specific effects of EA on DC maturation. Sex differences in glucocorticoid signaling and immune regulation are well‐documented: estrogen enhances DC activation and pro‐inflammatory responses, while androgens may amplify GR‐mediated immunosuppression [35, 36]. Furthermore, females exhibit heightened HPA axis reactivity, leading to differential corticosterone dynamics [37, 38]. These biological variations suggest that EA's efficacy in modulating the GR/GILZ pathway could diverge between sexes. Future studies comparing male and female responses to surgical stress and EA are imperative to elucidate sex‐dimorphic mechanisms. In addition, our study focused solely on mRNA levels of IL‐6 and IL‐1β, which may not fully reflect functional cytokine activity due to posttranslational modifications (e.g., proteolytic activation of IL‐1β). Future studies should incorporate protein‐level analyses (e.g., ELISA/Luminex) to evaluate critical cytokines such as IL‐12 and IFN‐γ, which are essential for antigen‐presenting cell functionality. The results provide novel perspectives on how EA might mitigate the negative impact of surgical stress on DC maturation by modulating the postoperative stress response and the GR/GILZ signaling pathway. However, GC‐mediated immunoregulation involves a highly complex network. For instance, GR can directly suppress NF‐κB or AP‐1 transcriptional activity independent of GILZ, thereby regulating pro‐inflammatory cytokine expression [39, 40]. Additionally, non‐genomic GC effects may synergistically modulate DC functional states [41]. Nevertheless, the reversal of DC maturation by RU486 and the coordinated changes in GILZ/GR expression suggest that GILZ‐dependent mechanisms likely dominate in this specific pathophysiological context. Future studies integrating multi‐omics analyses and conditional knockout models are warranted to fully dissect the spatiotemporal specificity of GC signaling in DCs.

Beyond mechanistic insights, our findings suggest EA as a promising adjunct therapy to counteract postoperative immunosuppression. By restoring DC maturation and inflammatory balance, EA could reduce infection risks and accelerate recovery, particularly in resource‐limited settings. Future clinical trials should evaluate EA's efficacy in patient cohorts, integrating immune monitoring (e.g., DC phenotyping, cytokine profiling) to validate translational potential.

## 5 Conclusion

The present study found that surgical stress under general anesthesia inhibits the immune maturation of DCs. EA mitigates the surgical stress response by suppressing the overactivation of the HPA axis, leading to reduced levels of CORT and ACTH post‐surgery. EA also enhances the expression of CD40, CD86, and MHC‐II on DC surfaces by modulating the GR/GILZ signaling pathway, thereby promoting DC maturation. Furthermore, the immunomodulatory effects of EA were found to be acupoint‐specific.

## Author Contributions

**Mengting Jiang:** data curation, investigation, validation, writing – original draft. **Caixia Liu:** writing – original draft. **Yinzhou Zhang:** formal analysis. **Sibei Li:** formal analysis. **Yuhui Li:** conceptualization, funding acquisition, writing – review and editing. **Chengcheng Zhou:** conceptualization, investigation, project administration, writing – review and editing.

## Conflicts of Interest

The authors declare no conflicts of interest.

## Data Availability

Data used to support the findings of this study are available from the corresponding author upon request.
